# P02.05. Clinical practice outcomes of an individualized integrative intervention for chronic pain: results from the BraveNet PBRN

**DOI:** 10.1186/1472-6882-12-S1-P61

**Published:** 2012-06-12

**Authors:** D Abrams

**Affiliations:** 1 University of California, San Francisco, San Francisco, USA

## Purpose

Chronic pain is one of the main conditions for which patients seek integrative medicine care. Many integrative medicine centers rank chronic pain as the complaint that they treat most effectively. We evaluated the impact of an individualized integrative intervention for chronic pain.

## Methods

Patients seeking their initial treatment for chronic pain at one of the nine centers in an integrative medicine practice-based research network were eligible. Enrolled patients received a personalized integrative medicine intervention over the 24-week study period. In addition to the Brief Pain Inventory (BPI), participants also completed the SF-12, PSS-4, CES-D 20 and various numerical rating scales. Repeated measures analyses were used to assess data from baseline, week 6, week 12 and week 24 visits.

## Results

252 participants completed the 24-week study. Participants were predominantly female (74%) with an average age of 49.8 years and 8.6 years of chronic pain. Pain was mainly in the axial skeleton, shoulders, knees and hips. The integrative intervention included combinations of acupuncture, massage, chiropractic manipulation, yoga, supplements (especially vitamin D3 and fish oil) and others. At baseline, median BPI Severity and Interference scores were both 5 (5=moderate, scored on 0-10 scale). By 24 weeks, BPI Severity decreased 20% to 4 (p=0.0006) and Interference decreased 40% to 3 (p<0.0001). Where 52% had symptoms suggestive of depression at baseline, only 35% did at week 24 (p<0.0001). Both the mental and physical components of the SF-12 improved significantly (p<0.0001) as did perceived stress (p<0.0001), sleep (p<0.006), fatigue (p<0.0001) and sense of control (p<0.0001).

## Conclusion

An individualized integrative medicine intervention successfully decreased chronic pain in participants with long-standing symptoms. Consistent with the suggestion that integrative interventions frequently impact on more than one endpoint, significant and consistent improvement was also documented in symptoms of depression, stress, and fatigue with positive effects on quality of life and sense of control.

